# Supplementary material for: The interactome of intact mitochondria by cross-linking mass spectrometry provides evidence for coexisting respiratory supercomplexes
Source: Mol Cell Proteomics. 2017 Dec 8;17(2):216–32. doi: 10.1074/mcp.RA117.000470 (PMC5795388; doi:10.1074/mcp.RA117.000470)
Supplement: Supplemental Data [file supp_RA117.000470_133922_0_supp_23978_fzffwf.zip › spectra_annotation/mito_DR_spectra_annotation/271-1-76-1-24-1.pdf]

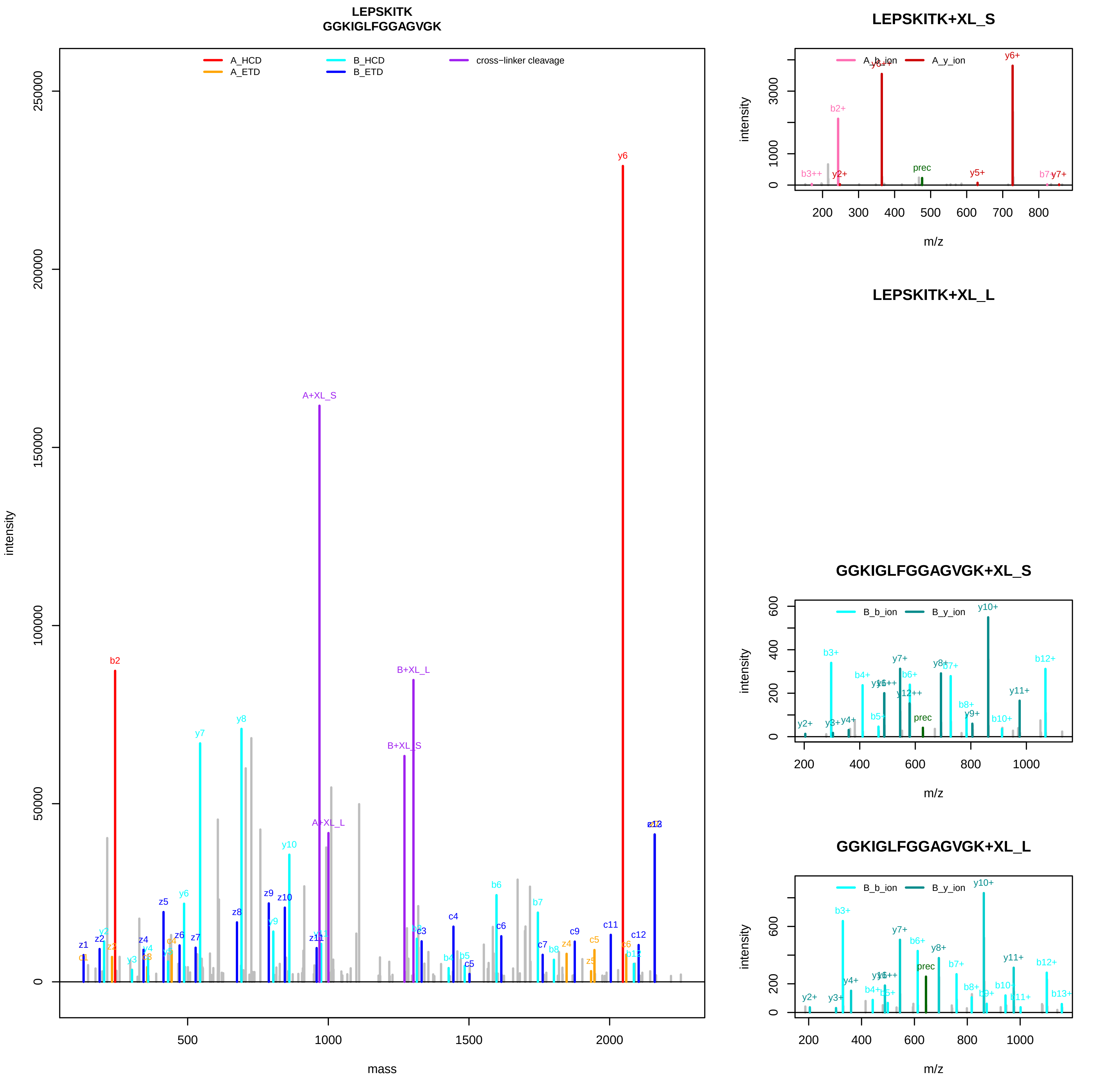

LEPSKITK
GGKIGLFGGAGVGK
A_HCD
A_ETD
B_HCD
B_ETD
cross−linker cleavage
intensity
mass
0
50000
100000
150000
200000
250000
500
1000
1500
2000
y6
A+XL_S
B+XL_L
B+XL_S
A+XL_L
b2
y8
y7
y10
b6
b7
LEPSKITK+XL_S
A_b_ion
A_y_ion
y6+
b2+
prec
y5+
m/z
0
1000
3000
200
300
400
500
600
700
800
LEPSKITK+XL_L
GGKIGLFGGAGVGK+XL_S
B_b_ion
B_y_ion
y10+
b3+
b12+
y7+
y11+
0
200
400
600
800
1000
GGKIGLFGGAGVGK+XL_L
